# Fair payment and just benefits to enhance diversity in clinical research

**DOI:** 10.1017/cts.2021.816

**Published:** 2021-07-14

**Authors:** Barbara E. Bierer, Sarah A. White, Luke Gelinas, David H. Strauss

**Affiliations:** 1 Multi-Regional Clinical Trials Center of Brigham and Women’s Hospital and Harvard, Cambridge, MA, USA; 2 Department of Medicine, Harvard Medical School, Boston, MA, USA; 3 Division of Global Health Equity, Department of Medicine, Brigham and Women’s Hospital, Boston, MA, USA; 4 Advarra, Columbia, MD, USA; 5 Columbia University Vagelos College of Physicians and Surgeons, New York, NY, USA

**Keywords:** Clinical trials, payment, compensation, diversity, inclusion

## Abstract

Routine, nonmedical and ancillary medical costs associated with participation in clinical research create barriers to enrollment for economically disadvantaged individuals. To the extent that race, ethnicity, and gender are linked to SES, such barriers impact efforts to diversify clinical research enrollment. But payment policies and practices often reflect the longstanding and singular concern that payment to participants will bias decision-making and compromise informed consent. We argue that this concern must be viewed in a larger ethical context in which the untoward consequences for the individual participant and for the broader research enterprise are considerable when either inadequate or no payment is provided for expenses incurred (“reimbursement”) and time committed (“compensation”). Fairness in payment and protection from undue influence of payment on the informed consent process are important but distinct ethical considerations. Fundamentally, approaches to payment that leave participants financially worse off as a consequence of taking part in research are inherently unjust as they have a differential impact on recruitment and retention based on socioeconomic status. Sponsors, funders, investigators, and IRBs must be cognizant of the impact of inadequate payment on clinical trial inclusion of historically understudied groups. We address practical and fair payment strategies to advance inclusion, the additional barrier of ancillary medical costs, and potential unintended consequences of payment.

## Introduction

While paying participants is not uncommon in research, there has been longstanding concern among sponsors, institutions, institutional review boards (IRBs) (alternatively termed research ethics committees), and investigators that payment to research participants will bias prospective participants’ decision-making and compromise their voluntariness—a circumstance referred to as “undue inducement” [1,2] within the U.S. regulatory lexicon [3]. In recent years, a re-consideration of this concern has emphasized distinct ethical considerations as they pertain to three categories of research payment: reimbursement for expenses; compensation for time, effort, and burden; and incentives to encourage participation [2,4]. We support this approach and further assert that the practice of nonpayment or underpayment of research participants for expenses incurred (“reimbursement”) and time and burden (“compensation”) introduces ethical concerns both at the level of the individual participant and for the larger research enterprise.

Ethically sound payment practices can ensure that the individual research participant will not be left worse off financially by having to assume nonmedical, study-related expenses. This position is enjoined by the Council for International Organizations of Medical Sciences (CIOMS) guidelines that describes its rationale simply: “participants should not have to pay for making a contribution to the social good of research” [5]. In the US, the FDA [6] and the Office for Human Research Protections [7] have each issued guidance supporting payment for research participation. Such costs include transportation, meals, child- and elder-care, and, for some, housing costs. Approaches to payment that respect the participants’ time and effort do not risk undue inducement and may be considered *fair* payment [2]. For the individual participant, fair payment minimizes financial burden and optimizes benefit in alignment with the notions of nonmaleficence and beneficence.

The practice of *not* paying (or underpaying) research participants also has undesirable implications of particular relevance to justice and to efforts to promote inclusion and diversity in clinical research [8,9]. For example, there is evidence that the costs incurred by participants in clinical trials represent a barrier to the enrollment of individuals of lower socio-economic status (SES) [10–13]. Inasmuch as some clinical trials provide access to novel, experimental, and often state-of-the-art treatments for an unmet medical need, financial barriers exacerbate health care inequity in the near term. Individuals, often from underrepresented and/or lower SES groups, who cannot afford the costs of participation are understudied, and therefore these groups are deprived of the long-term benefits of research [9–14].

Individuals of lower SES are less likely to be able to afford the time and expense of uncompensated research participation; payment may encourage individuals to participate who otherwise would not be able to afford the associated costs.[2,4,14]. Such costs include time away from income-generating activities and parenting duties and the time and expense of travel to and from the research site, among others. The loss of income is particularly noteworthy—and burdensome—for hourly employees and those at the lower end of the salary scale. Other costs of clinical trial participation, such as those associated with medical care, ancillary care, and post-trial treatment, represent significant additional barriers that we consider further below [8].

Historically, underrepresented populations in research (such as racial and ethnic minorities and older individuals) are often overrepresented in low-income groups [15,16]. Other understudied groups (e.g., children and adolescents, women, rural populations) require special consideration in relation to the required costs and effort of research participation. If there are financial barriers to clinical trial participation, these may contribute to the well-documented failure of the demographics of clinical trial participants to reflect the population at large or the demographics of those affected by the disorder in question [10,14]. Attention to policies and practicalities of payment that address these barriers may serve to promote more representative inclusion in clinical trials. In this way, approaches to fair payment support access and ensure that medicinal products are tested in the populations likely to use them.

## Strategies and Practices to Promote Fair Payment

Much scholarly work has explored the ethical foundations of payment and helped frame concerns about both exploitation of participants (when fair payment is not provided) [17,18] and undue inducement (when offers of payment may introduce incentives that bias decision-making) [1,2,19]; other literature has addressed the lack of diversity among participants in clinical trials and documented impediments to change [14,20]. However, little attention has focused on the logistics of ethically sound payment strategies in the planning and implementation of a clinical trial. Here we discuss the practical implications of payment, with particular attention to how they impact individuals of lower SES. We develop specific considerations regarding the design, evaluation, and provision of payment for research participation; discuss risks of payments and their mitigation; counsel sponsors and funders to provide payment; explore attitudes, education, and actions of research review and oversight committees to approve payment; encourage institutions and investigators to create mechanisms to pay appropriately; and, finally, summarize additional logistical concerns.

## Designing, Evaluating, and Offering Payment for Research Participation

Payment strategies should be informed by input from participants, participant groups, and where relevant, their families or communities. In consideration of both the ethics and the logistics of payment to research participants, payment should be partitioned into three categories: (1) **reimbursement** for expenses incurred as a result of participation, (2) **compensation** for time and effort related to research participation, and (3) **incentive** payments to encourage participation, retention, and study completion [2].

1. ***Reimbursement*** of expenses

Whether and how much reimbursement is offered will depend, at least in part, on the study budget and funding; therefore, study planning must allocate sufficient funds to reimburse individuals for reasonable out-of-pocket expenses recognizing the unfair burden otherwise placed on the economically disadvantaged. Such costs associated with participation should be anticipated and explained during the consent process. The nature of eligible and reimbursable expenses should be detailed. If a participant requires the help of a parent, guardian, or caregiver to travel to the site, these costs should be considered reimbursable expenses. Any limitations on reimbursement should be determined in advance and be made explicit (e.g., mileage allowance, maximum reimbursement for hotel or parking, coach airfare) in the consent and/or accompanying materials. Of particular importance is information about whether, and the extent to which, the costs of ancillary care (e.g. medication to treat study-associated nausea or vomiting) will be the responsibility of the participant or whether such expenses will be covered or reimbursed. This is discussed further below.

There are a variety of methods to provide reimbursement, including reimbursement for submitted expenses, providing an average amount for estimated expenses, and preloaded debit cards. The first method requires participants to document all incurred expenses; this is, however, sometimes challenging as some expenses are paid in cash and may not have accompanying documentation, and some receipts may be misplaced. Notably, tracking, filing, submitting, approving, and reconciling payment are burdensome not only to the participant but also to site staff.

As an alternative to expense-based reimbursement and its detailed accounting, the average expenses for participation may be estimated and offered to participants. The amount of such payment will vary by site and by country in multisite and multinational research, as the costs and expenses of participation will vary, as well as by distance traveled. As discussed below, however, the participant remains responsible for maintaining documentation of expenses, lest the reimbursement be considered income and subject to tax.

Reloadable, auditable, prepaid debit cards are convenient, incur no delay for the participant, can automate the classification of taxable and tax-exempt expenses, and can be outsourced. If access to debit cards is outsourced, participants should understand that their personal information will be shared outside the institution. Pharmaceutical sponsors and institutions should clarify who is responsible for arrangements and for responding to participant concerns.

Every effort should be made to pre- or re-pay expenses quickly, as some participants will not be able to outlay necessary funds nor have sufficient credit to carry, or cash to pay off, a balance. Delay in reimbursement will create greater hardship for individuals of lower SES, and efforts to advance payment or direct pay expenses (e.g., through prepayment or use of a debit card) will have advantages in recruitment and retention. If possible, participant should be asked about their preferences for the form and process of reimbursement.

Any planned reimbursement, policy, or practice should be communicated in advance, including in materials provided to the participant and provided in language understandable to that person, and be clear as to whether, when, and how exceptions will be considered. The process for review and approval or rejection of exceptions (and process for appeal, if any) should be described in the policy and participant-facing materials. Reimbursements for reasonable expenses are generally considered tax-exempt, but country-specific tax law will apply. Exceptions, if any, should be disclosed to the participant.

1. ***Compensation*** payment for time- and research-related burdens

Fair compensation for time and burdens of research may be permitted and even encouraged, particularly as the costs for participation (and therefore loss of wages from lost time at work) differentially impact traditionally underrepresented and underserved populations. Further, offers of compensation respect the time contributed by participants. Depending on the risks and anticipated benefits of study participation, failure to offer payment can be viewed as exploitative and will be felt most acutely by those who can least afford it. The burdens of research may include time and effort required and associated inconvenience (e.g., fatigue and minor physical, emotional, or psychological discomfort.) If compensation is offered, a fair rate should be determined in advance, considering time expectations and burdens of participation, equivalent for and offered to all participants at a given location, not based on actual individual wages or earning capacity, and paid in a timely fashion.

There is no generally accepted methodology for determining compensation. Different methods for determining appropriate amounts of compensation for *time* have been suggested, including minimum wage or some small multiple thereof, or a rate equivalent to non-research, unskilled employment [4]. For example, the guidance given in CIOMS states, “The amount of compensation should be proportional to the time spent for research purposes and for travel to the research site. This amount should be calculated using the minimum hourly wage in the region or country as a reference value” [5]. Whatever methodology is chosen can be adjusted for location, as fair compensation will differ depending upon the country, region, and urban versus rural locations. Given the variability of clinical benefit from research participation, and in recognition of the participants’ contribution to research, offering--at a minimum--a working wage, minimizes financial harm for those least able to bear that expense and eliminates what might constitute a disincentive for participation.

Uniform methods for determining additional compensation for the *burdens* of the research have not been made available [21], nor are there empirical data on participant expectations. Some institutions have established guidelines for compensation for specific procedures (e.g. blood draw, MRI) in part to assure fairness across studies, although these rates are not often shared across institutions [21]. Research procedures and anticipated burdens should be delineated, compensation amounts suggested, and recommendations reviewed and approved by the IRB with local knowledge. Optimally, local and participant community input into the amount and method of compensation will be sought.

If sponsors, research sites, and/or IRBs intend to set payment amounts for specific research procedures, these amounts should be anchored in standard operating procedures (SOP) to account for variation while promoting consistency. Such guidelines should be informed by the participant perspective, approved by the IRB, respectful of the voluntary nature of the participant’s contribution to the research, and sensitive to the elective nature of the procedure when not otherwise medically necessary. For instance, a standard institutional policy may set payment at $15.00 for a single blood draw, and the SOP may specify other important considerations: that payment is provided for any venipuncture attempt, whether successful or not, and only when incremental to medical care. Such guidelines should also anticipate circumstances when procedures are cancelled and may specify reasonable compensation for the subject who has sacrificed a workday and traveled for the procedure.

Compensation for time and the burdens of research should be determined in advance; based on local, not national or international standards; reviewed and approved by the IRB; and communicated to the potential participant. When a single IRB reviews research for a multicenter trial, the reviewing IRB should acknowledge local practices and guidelines when these reflect community standards or differ from their own. Compensation should be made available at reasonable intervals as time and burdens are experienced, not held until the end of study. The expectation that participants be compensated for time and burdens incurred does not prevent consideration of a “completion” bonus, that is, an additional, reasonable payment for completing all parts of the protocol [22]. Individuals who withdraw or are withdrawn should be compensated up to and including the time of withdrawal. Of course, compensation may be withheld if a participant fails to comply with the research procedures through willful neglect or noncompliance. The terms of compensation, including its dependence upon participant compliance and the timing of payments relative to study activities, should be communicated in advance and in writing, potentially in the form of an IRB-approved, participant-facing, health literate document.

The question of whether payments may be offered in amounts proportional to the risk of harm is the subject of ongoing debate. Some sponsors and institutions opt for, and some IRBs approve, compensation to offset the risks of research, and some guidance allows for it [23]. Others maintain that paying individuals more to assume greater risk of harm is inherently problematic, especially for those who are financially disadvantaged.

1. ***Incentive*** payments

Incentive payments, payments beyond reimbursement and fair compensation, are intended to increase study recruitment, retention, and completion but involve more complex ethical considerations. Incentive payments are intended to motivate participation and retention and may impact individuals’ decision-making according to the perceived value of the incentive, based, at least in part, on SES. Because incentives go beyond what can be considered fair reimbursement and compensation, additional safeguards may be warranted to ensure that financial considerations do not override other factors related to protocol adherence and participant safety and well-being [9].

It is fair and defensible to adjust payment for participation based on trial requirements inclusive of a reasonable completion bonus. For instance, three visits may be necessary to answer a trial-related question. While expense reimbursement and compensation may be the same for each of the three visits, incentive payments may differ or an incentive payment may be offered for the third and final visit; that visit may be “valued” at a higher rate, as the individual’s participation is only useful to the research if the participant completes all three visits. It would not, however, be appropriate to withhold the entire amount until the end of study. The IRB should review and approve any proposed incentive payment and any safeguards to ensure voluntary informed consent to enrollment and ongoing participation. IRBs and investigators should remain attentive to the possibility that payment may impact a participant’s decision to enroll or remain in a trial when it is clinically contraindicated, and that this may differentially impact those of lower SES. Incentives that would encourage a participant to conceal important clinical information in order to access or remain in a study would not serve the participant’s best interests or the aims of the research—and would raise ethical and scientific concerns. Where possible, objective inclusion/exclusion and study discontinuation criteria and periodic review of medical records may help to mitigate these risks.

## Ancillary Costs of Participation

There are potential costs to participation in addition to the direct reimbursable expenses discussed earlier, the most significant of which are referred to as the “routine costs” of clinical trial participation such as ancillary medical care, physician and hospital visits, and treatment of research-related injury. Payment for routine costs globally is a complex matter. In the US, Medicare provides coverage for routine costs of qualifying clinical trials as well as “reasonable and necessary items and services used to diagnose and treat complications arising from participation” [24], and private insurance providers were required by the Affordable Care Act to provide similar coverage, from 2014 onwards, for the prevention, detection, and treatment of cancer and other life-threatening diseases [25]. Excluded from payment under federal law were the costs of other clinical trials as well as Medicaid recipients. A patchwork of state laws resulted [26], leaving the majority of Medicaid beneficiaries, including low income and populations underrepresented in research, unable to access clinical trials. Medicaid coverage for qualifying clinical trials will begin in 2022 [26]; unfortunately, while critically important and potentially offering direct benefit, qualifying clinical trials represent only a proportion of clinical trials. For those trials not covered by federal laws, and for individuals with no health insurance, the lack of payment for such routine costs (including research-related injury) remains a significant disincentive to participation.

At a minimum, the informed consent document and process should clearly present these additional costs of participation. As a matter of justice [27,28] and of scientific integrity, however, this is an insufficient solution. In the short term, health insurance could be provided to participants for their role, paid by either sponsoring pharmaceutical entities, institutions, and non-profit funders, or cost waivers or debt-forgiveness could be engineered by sites. In the long term, in the absence of national health insurance, sponsors, investigators, patients, and their advocates should call on the federal government to expand its coverage and provide a safety net for routine and other costs. Inroads into understanding and correcting health inequities will not be achieved without appropriate representation in research.

## Non-financial Benefits

Individuals  who choose to participate in clinical research, including those from lower socio-ecomomic status, do so to obtain benefits beyond payment. While a theoretical argument can be made to justify participation as “work” by research volunteers [29], rarely is compensation significant, even for healthy volunteers participating in Phase 1 studies [30]. It is therefore important to optimize, during the planning, design, and conduct of the trial, nonfinancial benefits for the participant, the value of which will vary by participant and participant group. Nonfinancial benefits may uniquely offer benefit to the uninsured and underinsured and others for whom access to routine medical care is affected by SES. Examples of those benefits include access to (1) rigorous diagnostic assessment, disease management, and/or counseling; (2) health services unrelated to the research [5], (3) diagnostic and/or genetic testing; (4) post-trial access to care or to continued access to an investigational product; (5) devices, often for free, such as a glucose monitor or hearing aid; (6) advice, such as nutritional counseling or an exercise program; (7) small gifts (e.g., a pedometer) or small tokens of appreciation; and (8) appreciation for the contribution to science and public health. The contribution to science translates into public benefit to the community including awareness, knowledge, and access.

## Further Considerations of Payment

The impact and consequence of payment will differ depending on income, locale, dependency on government entitlements, and SES, and, therefore, especially impact recruitment and retention of individuals of lower SES.

### Risks to Privacy and Confidentiality

Investigators, institutions, IRBs/RECs, and sponsors should recognize that submission of personal identifying and financial information may incur incremental risks to privacy and confidentiality for the participant. Privacy and confidentiality risks exist for all volunteers in the US, but there are additional risks to people of lower SES. In the US, for instance, many institutions require a Social Security Number or Taxpayer Identification Number (SSN/TIN) in order to provide compensation (e.g. check, money order, automatic deposit) to a participant. These requirements are problematic for those who choose not to share their identifying information or for those that do not have an SSN/TIN and may deter some individuals from participating or, at a minimum, from being fairly paid for participation. Depending on jurisdiction and amount, the institution will be required to disclose personal information to the government, a problematic consequence for some individuals such as undocumented immigrants. In addition, institutional business offices may inadvertently disclose health information in financial records or on the payment, for example, when the condition being studied or the protocol title appears on the check. Steps to mitigate such risks should be addressed at the time of study planning and IRB review. The participant should be made aware of foreseeable risks.

### Risks of Participant Eligibility to Entitlements

In any setting, additional unanticipated consequences of payment to the participants should be considered. In the US, for instance, Supplemental Security Income (SSI) is a US government means-tested welfare program that provides cash assistance and health care coverage to people with low income and to those with limited assets who are older than 65, blind, or have a disability. SSI and Medicaid currently exclude the first $2,000 of income per year in the calculation of eligibility, but only for certain clinical trials studying treatments for rare diseases and not for other diseases [31]. The Supplemental Nutrition Assistance Program (SNAP) program, for example, may reduce benefits by monthly income [32]. Provision of payment for research participation could jeopardize eligibility for federal and state program as eligibilities differ by family size, threshold amounts, and other considerations. These are real risks to individuals that should be disclosed and discussed in advance with the participant.

### Potential Tax Consequences of Payments

Participants should be alerted to the fact that there may be tax implications of payments for research. Reimbursement for documented expenses is not generally taxable, and participants should be advised to maintain receipts for expenses. Notably, however, reimbursement of estimates of expenses may be considered taxable unless the payee can provide evidence that actual expenses equalled or exceeded the amount paid. In many countries including the US, compensation and incentive payments incur a tax liability for the participants. US tax law requires reporting of income by the payer (if greater than $600 annually) and by the “independent contractor” (e.g., the participant, with no de minimus) and should therefore be considered taxable income. While the specific tax consequences are not the responsibility of the IRB (nor the expertise of the authors), potential implications of payment should be communicated in advance and included in advance or at the time of enrollment.

### Risk of and Compensation for Research-related Injury

Reimbursement for expenses for research-related injury is highly variable by protocol, institution, state, and country and should be considered separately from payment for research participation. While compensation regulations and policies for research-related injury vary by country [33], US federal regulations require only that participants be informed as to whether medical or other care, or compensation for injury, will be available in studies that pose greater than minimal risk, and they forbid exculpatory language [3]. They do not, however, mandate compensation for research-related injury. The consequence on the individual should be considered and discussed, as participants may suffer economic and other harms as a result of research-related injury, and efforts should be made to compensate individuals for those harms when they occur. It may be possible to purchase medical insurance for participants who are not otherwise insured, or to offer insurance for all participants, for research-related medical care.

### Logistical Convenience

Many sites consider the practice of arranging payment for participants to be unduly burdensome. Institutions are advised to develop payment policies and routinized systems for payment that are, nevertheless, sufficiently flexible that they accommodate participant needs (e.g. prepayment) and wishes (e.g. check, debit card, or direct deposit) and that comply with local tax law. When developing the remuneration approach, every effort should be made to simplify the process and avoid detailed accounting, processes, and paperwork. An estimate of travel costs, for example, may be sufficient for reimbursement rather than requiring odometer readings and toll receipts for each trip. The time required on the part of both the participant and the research staff should be weighed against the incremental benefit in precision.

Investigators and institutions may wish to consult with the sponsor of a study to develop methods that work for all parties. Some commercial vendors have developed automatic payment processing systems for sites—including automated payment for participants—that appear to allow efficiency, audit and reconciliation, transparency, and international functionality. Secure, reliable debit card technologies typically incur a fee for service.

## Conclusion

Participants should not be further economically disadvantaged by agreeing to volunteer in clinical research. Sponsors, investigators, and IRBs should consider the impact of study payment, or non-payment, on the recruitment and retention of study participants with attention to SES. During the consent process, investigators should be attentive to how payment considerations affect participant decision-making. Planning of a trial should include identification and delineation of reimbursable expenses and of fair compensation for time and burden. Incentives for participation and completion bonuses are challenging and should not be so high as to deter individuals from withdrawing from a study. IRBs should review all planned payments and their justification, the specifics of the proposed disbursement methodology and timing, and the description in the informed consent document and any accompanying explanatory texts intended for participants. However, IRBs should consider payment as an acceptable and appropriate practice, and one that not only serves to respect participant contributions but may impact efforts to enroll and retain groups who are traditionally underrepresented in research. Sponsors and funders should anticipate participant expenses and allow those costs to be incremental to the per-patient or study budget. Institutions should provide oversight to ensure that equivalent research procedures are valued equivalently, and that participants are paid either in advance or promptly. Processes should be established to minimize burden for investigators, study staff, participants, and their caregivers.

Risks and limitations of payment should be analyzed, mitigated if possible, and explained in plain language to the participant. Whether payment is considered taxable income or results in loss of entitlements will depend upon state, country, and circumstance. Routine costs and potential costs of research-related injury, if not a qualifying clinical trial and/or covered by Medicare, private insurers, and (soon) Medicaid, should be identified, and additional insurance, debt-forgiveness, or payment provided. Nationally and internationally, the clinical research stakeholders as well as patients and their advocates should champion for coverage.

In addition to financial consideration, participation in research offers non-financial benefits for research participants that vary according to the nature of the research and participant group. Benefits of research participation may accrue to the individual, the family, and/or the community. Enriching the experience of research participation and acknowledging participant contributions can advance diversity and inclusion in recruitment and retention, fosters good will, and results in knowledge that is applicable and generalizable, the objective of the research enterprise itself.
